# Dynamic prediction of survival via Landmark method using the asthma prevention trial in young children

**DOI:** 10.1371/journal.pone.0293796

**Published:** 2023-11-30

**Authors:** Nosheen Faiz, Atal Khan Gardiwal, Muhammad Asif Khan, Soofia Iftikhar

**Affiliations:** 1 Department of Statistics, Abdul Wali Khan University, Mardan, KP, Pakistan; 2 Social Policy, Evaluation, Analytics and Research, UNICEF, Kabul, Afghanistan; 3 Qatar Mobility Innovations Center, Qatar University, Doha, Qatar; 4 Department of Statistics, Shaheed Benazir Bhutto Women University, Peshawar, KP, Pakistan; Bindura University of Science Education, SOUTH AFRICA

## Abstract

This paper focuses on the applications of Landmark method for obtaining dynamic predictions of survival by using Landmark approach to the data of asthma prevention trial in young children. This work focuses on the different ways to model recurrent events by considering various time scales according to how subjects in the dataset experienced multiple events. Landmark models can be used to dynamically estimate the effect of treatments effects whilst also taken into consideration the history of previous asthma attacks. Our analysis show that the treatment effect should be modelled with a time varying effect and the effect of the previous attack reduces with the passage of time.

## 1 Introduction

Time-dependent marker information collected from a patient’s follow-up are used by Dynamic prediction to construct an improved and more accurate estimates of their survival probability [1]. Conventional prediction model, for example, the Cox model, however, can not be applied at a specific prediction time during patient’s follow-up. As the Cox model can not account for long term survival. Although, if we have time varying effects and non-proportional hazards models, these models do not account for conditional survival (i.e., probability of surving 5 years given that patient has already survived 3 years). In the literature, existing methods for dynamic prediction are the Joint models and Landmark. For Joint modeling a model is specified for the longitudinal marker process and a model for survival outcome. These two models are then linked by a function [2, 3]. Estimation of model and calculation of the conditional survival probabilities can be quite time consuming [3, 4]. Therefore, computational complexity and misspecification adversely affects the performance of Joint modelling. Landmarking is implementated straight away, however, it depends on a proportional hazards model and specification of a sequence of survival models for the subsample of patients still at risk at prediction times during follow-up, which is called landmark times [5]. A Cox proportional hazards model is employed in classic Landmarking. This technique does not need the specification of distribution of the stochastic marker process in time. This property makes Landmarking more attractive as compared to Joint modeling. Prediction accuracy of the Landmarking is affected by misspecification [6–8]. Authors in [6, 7], have suggested Landmark analysis and Joint models for dynamic prediction of survival probabilities using longitudinal and time-to-event data. Dynamic prediction can be utilized to obtain functional form to link longitudinal and time-to-event processes. Thus, the distinctive events and calibration can also be measured. Van Houwelingen and Putter [9] have made comparison between new Landmark methodology and multi-state modelling. Generally, both these approaches provide similar outcomes, however, the Landmark method does not require complicated modelling and the predictions can be easily obtained. Alternatively, the Landmark method has no intuitiveness in biological processes while multi-state model can be utilized in this particular area. Several researchers have implemented the Landmark approach for dynamic prediction of survival considering time-to-event data, see for example, [1, 10–12]. The Landmark approach has been further extended by introducing different models with parametric effects of the Landmark point and fitted by maximizing pseudo-likelihoods which expand the partial log-likelihood. According to Parast et al., [13] while dealing with specific disease, one may use onset time of an observable short term event to predict the long-term survival as it may highly correlates with a sub-types of disease. This can be implemented by using Landmark Cox model, which considers the proportional hazards model to be fitted at each Landmark point for the residual life. For this purpose, the predictive performance is quantified by using the time-varying accuracy measures and inference about these accuracy measures are drawn on the basis of re-sampling based procedures. In Landmark analysis, the effect of time-dependent covariate is estimated based on the Landmark point considering the covariate value which may change after the Landmark point since, the covariate is time-dependent which is essential for finding relation between the coefficients in the Landmark analysis for time-dependent variables coefficient and baseline hazard of Cox regression for time-dependent variables. In this work, we focus on Landmark to obtain dynamic predictions of survival by applying Landmark approach to the data of asthma prevention trial in young children. Such predictions provide a basis for making precise decisions regarding the continuation or stopping of a particular treatment or medicine given to the patients considering their prognosis at different time points during the follow-up period. The Landmark is the best method used to obtain dynamic predictions based on time-to-event data.

The contribution of the paper is as follows: We aim to focus on utilizing the Landmark approach to obtain dynamic predictions of survival in the context of an asthma prevention trial in young children. It demonstrates how this approach can be effectively applied to analyze time-to-event data and generate predictions. Also, it showcases the significance of dynamic predictions derived from the Landmark approach. It highlights how these predictions serve as a basis for making precise decisions regarding the continuation or cessation of specific treatments or medications for patients. By considering patients’ prognosis at different time points during the follow-up period, informed treatment decisions can be made.

## 2 Data description

The dataset used in this study is related to asthma prevention trial in young children. Children who have not yet experienced asthma attack but with high risk of asthma enter the study. The age for entering the study was 6 months and they were followed for 18 months. The data contain 1037 observations with 7 variables. The number of observations does not depict the number of children involved in the study. The reason is that we do not have a single event (asthma attack) for an individual as all of them have experienced different numbers of events during the study period. It can be observed from the data that different individuals have experienced different number of asthma attacks meaning that there are recurrent events rather than a single event for each individual. There are a total of 232 children involved in the study. During the study period, all the children had experienced 819 asthma attacks. Hence, the average number of asthma attacks experienced by each child is 3.53. There are seven variables in the data. They are; the patient identity number (id.w), treatment either placebo or medication (trt.w), starting time of follow-up in days (start.w), stop time which shows the time of the occurrence of an event in days (stop.w), status of patient (st.w) at a specific time during the study (censored—“0” or event—“1”), the number of entries (nn) in data for each patient and the last variables gives information regarding the occurrence of first event (fevent). The children with high risk of asthma were randomized to a medication or a placebo. Out of total 232 children, 113 of them were randomized to medication while 119 to placebo. This dataset is available at https://data.world/muasifkn/asthma-prevention-trial-in-young-children. Table 1 provides the summary of the number of children experiencing the number of asthma attacks.

**Table 1:** Summary of the number of children experiencing different number of asthma attacks.

No. of asthma attacks	No.of children experiencing different No. of asthma attacks	Percent	No.asthma attacks	No.of children experiencing different No. of asthma attacks	Percent
1	232	100	21	1	0.431
2	145	62.5	22	1	0.431
3	100	43.1035	23	1	0.431
4	72	31.0345	24	1	0.431
5	57	24.569	25	1	0.431
6	45	19.397	26	1	0.431
7	37	15.948	27	1	0.431
8	30	12.931	28	1	0.431
9	20	8.621	29	1	0.431
10	19	8.189	30	1	0.431
11	11	4.741	31	1	0.431
12	9	3.879	32	1	0.431
13	7	3.017	33	1	0.431
14	4	1.724	34	1	0.431
15	3	1.293	35	1	0.431
16	2	0.862	36	1	0.431
17	2	0.862	37	1	0.431
18	2	0.862	38	1	0.431
19	2	0.862	-	-	-
20	2	0.862	-	-	-

## 3 Landmark approach

Anderson et al. [14] introduced the Landmark concept to deal with the time dependent covariate in survival models. If the proportional hazards assumption is violated the simple Cox model can still be able to provide plausible prediction of survival up to certain horizon (*h*_*hor*_). In order to get dynamic predictions, fitting simple Cox model might be erroneous therefore, the Cox model can not take up dynamic differences in any case. To obtain predictive probabilities for dynamic predictions of the survival, the Landmarking approach can be used [15]. Selecting individuals at risk at a specific time point and use the information available at that time point for making predictions, this is how the predictive probabilities can be obtained from the data and this process is known as landmarking.

In the following subsections, we introduce two key variations of the Landmark approach: the Simple Landmark Model and the Landmark Supermodel. These models serve as integral components of our research methodology, each offering distinct advantages and considerations in predicting survival outcomes.

### 3.1 The simple Landmark model

In the Landmark approach the fundamental rule is the splitting of dataset into short time period to obtain the Landmark points within a sequence starting from 0 (the first Landmark time point) with interval. Each point that splits the dataset is known as Landmark (prediction) time point. The Landmark point is denoted by *t*_*LM*_. A certain horizon (*t*_*hor*_) is specified and simple Cox model is fitted on the interval (*t*_*LM*_, *t*_*hor*_) in order to get a prediction of survival. Van Houwelingen in [16] argued that fitting such a model in the interval (*t*_*LM*_, *t*_*hor*_) with covariates having time-varying effect will still provide approximate estimates. In order to get a sliding Landmark dataset, it is necessary to apply truncation at Landmark point *t*_*LM*_ and administrative censoring at a horizon *t*_*hor*_ = *t*_*LM*_ + *w*. The sliding Landmark model for obtaining such a prediction is basically a simple Cox model

$$\begin{array}{c} h\left(t|X,t_{LM}\right)=h_{0}\left(t|t_{LM}\right)\text{exp}\left(X\beta_{LM}\right),\;t_{LM}\leq t\leq t_{hor} \end{array}$$ (1)

This model can be applied for all the individuals at risk at the assessment point or Landmark point *t* = *t*_*LM*_ and neglect any event after a certain horizon (*t* = *t*_*LM*_ + *w*). The estimates of both coefficient (*β*_*LM*_) and baseline hazard *h*_0_(*t* ∣ *t*_*LM*_, *w*) can be obtained by fitting a simple Cox model to the sliding landmark dataset.

### 3.2 The Landmark supermodel

Van Houwelingen [16], presented a prediction model used for a range of prediction times while focusing on the construction of a super prediction dataset. Initially, the prediction window “*w*” is fixed to get a super prediction dataset and landmark points or the points of assessment *s*_1_, ……., *s*_*L*_ are selected. After the selection of Landmark points, the prediction dataset for each Landmark point (*t*_*LM*_ = *s*_1_) is created by applying truncation and administrative censoring and all the datasets created for each prediction time point are combined into a single super prediction dataset.

Van Houwelingen [16] has used slightly different pseudo-likelihoods to fit models for *β*_*LM*_(*s*). Fitting a Cox model to a super prediction dataset with stratification on *s* is the first approach and the model is given by

$$\begin{array}{c} h\left(t|X,s\right)=h_{0}\left(t|s\right)\text{exp}\left(X\beta_{LM}\left(s\right)\right)\;for\;t\geq s \end{array}$$ (2)

where, *β*_*LM*_(*s*) = *f*(*s*)*θ* and *f*(*s*) is a set of basis function (contain constant, linear, and quadratic functions) and *θ* is vector of parameters. It is worth noting that regression parameters rely upon *t*_*LM*_ = *s* (the prediction time) while they do not depend on *t*_*i*_ = *t* (the event time). In the case of time-varying effect covariates, fitting a model for varying values of s is straightforward but the challenge is to get a model for *β*_*LM*_(*s*) and *h*_0_(*t* ∣ *s*) [16]. It is feasible in such a way to stack the Landmark datasets in practice and to extend pseudo likelihoods. *β*_*LM*_(*s*) can be modelled by maximizing the pseudo-log-likelihood given as follows,

$$\begin{array}{c} ipl\left(\beta_{LM}\right)=\sum_{i=1}^{n}d_{i}[\int_{0}^{t_{i}}X_{i}\left(s\right)\beta_{LM}\left(s\right)\psi \left(s\right)ds-\int_{0}^{t_{i}}\text{ln}(\sum_{t_{j}\geq t_{i}}\text{exp}\left(X_{j}\left(s\right)\beta_{LM}\left(s\right)\right))\psi \left(s\right)ds], \end{array}$$ (3)

where

*t*_*i*_ is an observation time

*d*_*i*_ is an event indicator

*X*_*i*_ is a vector of covariates

*ψ*(*s*) be a non-negative function (with *ψ*(*s*) = 0 if *s* > *t*_*hor*_) that specifies how the possible Landmark points are weighted in fitting models for *β*_*LM*_(*s*)

The estimate of the Landmark specific baseline hazard is given by

$$\begin{array}{c} {\hat{h}}_{0}\left(t_{i}|s\right)=\frac{1}{\sum_{t_{j}\geq t_{i}}\text{exp}\left(X_{j}\left(s\right){\hat{\beta }}_{LM}\left(s\right)\right)}\;for\;t_{i}>s \end{array}$$ (4)

## 4 Materials and methods

The dataset is modified in such a way that two new variables are added to the dataset where the two variables are derived from the same dataset. The first variable is derived by subtracting the variable i.e., “starting time” of follow-up from the variable “stop time” thus, the resultant variable is actually a gap time. This new variable is important in the sense that it is used as a time for fitting various models throughout this study. The second variable is obtained by providing a proper sequence to the events experienced by each individual that helps in modelling various numbers of events experienced by an individual during the study period and it also helps in plotting Kaplan-Meier curves. The different Kaplan-Meier curves are plotted based on the sequence of the asthma attacks experienced by each individual to observe the survival curves for the asthma patients randomized to either medication or placebo. For checking treatment effects and evaluating the importance of frailty term various frailty models are fitted on the dataset where the frailty models are based on the usage of various time scales either calendar time or gap time. The simple Cox model [17] based on calendar time and gap time is separately fitted to the data. Similarly, the simple Cox model [17] is applied to the data considering only the first event (the asthma attack) experienced by all the participants of the study where all other subsequent events are neglected. Eventually, the Cox frailty-calendar model and the Cox frailty-gap model [18] are applied to the data. Steps involved in the exploited Landmark method are given as follows,

1. For the implementation of Landmark technique the dataset is split to obtain the Landmark points within a sequence starting from 0 to 400 with interval of 5 days. It means the first Landmark time point would be 0 and the following Landmark time points would be 5, 10, 15, 20, …., 400, since, the distance between two Landmark time points are chosen to be 5.
2. In the second step a horizon is chosen to be 120 days. Hence, the total number of Landmark time points obtained is 81. It can also be put in such a way that total 81 datasets are created which can provide dynamic predictions at 81 different time point considering each of these datasets one by one.
3. For smoothing and simplification, the truncation technique is applied at each Landmark point and administrative censoring at horizon for obtaining sliding Landmark datasets.
4. In the final step the simple Landmark model (see Section 3.1) is implemented on each of the total 81 newly created datasets. Each model provides the estimates of coefficient for a specific Landmark time point. The independent variables in the model are *trt*.*w* (either treatment or placebo) and *cnts* (referring to the sequence of asthma attack experienced by each participant of the study). Also, the coefficients for both variables are plotted to show the effect of treatment and the associated risk related to the asthma patients.

However, fitting model 1 does not address the relation between *β*_*LM*_ and *t*_*LM*_ and it also ignores the overlap between different Landmark datasets. Therefore, to overcome this drawback, the super prediction dataset is created. The same sequence and prediction window is selected as it was used to get sliding Landmark datasets. The total 81 newly created datasets are stacked into single dataset called the super prediction dataset. The model is fitted to the super prediction dataset with stratification on the Landmark point which is basically the super Landmark model 1. The final task is to fit a super Landmark model 2 where this model provides the estimates by using unstratified analysis and the inclusion of Landmark term to the model.

## 5 Software and packages

For executing the various models, obtaining the results and comparison with other methods in this study R programming language is used https://www.rproject.org. R packages i.e., “survival” [19] and “dynpred” [20] are the two main packages used in the analysis. The “survival” package contains the core survival analysis routines, including definition of Surv objects, Kaplan-Meier and Aalen-Johansen (multi-state) curves, Cox models, and parametric accelerated failure time models [19]. The other package is “dynpred” [20] which contains functions for dynamic prediction in survival analysis. For splitting and combining data “plyr” package [21] is used. Similarly, “survminer” package [22] is applied to obtain lavish survival curves.

## 6 Experiments and results

In this subsection, we present a preliminary analysis that serves as the foundation for our subsequent investigations. The purpose of this analysis is to gain initial insights into the dataset and establish a basis for the more in-depth analyses conducted later in the study. We begin by providing a descriptive overview of the key variables and characteristics of the data. Next, we explore any notable trends or patterns that emerge, highlighting initial observations that could potentially influence our research findings. Furthermore, we discuss the limitations of this preliminary analysis and acknowledge the need for further examination. By conducting this preliminary analysis, we aim to lay the groundwork for a comprehensive and informed exploration of our research topic.

### 6.1 Preliminary analysis

The Kaplan-Meier plot for the asthma patients randomized to either medication or placebo based on the sequence of the asthma attacks experienced by each individual.

All the given Kaplan-Meier Survival Curves illustrate the survival probabilities on y-axis and time on x-axis. The green curve shows the survival for children randomized to medication while the red curve shows the survival for children randomized to placebo. Each plot compares the survival probability for those either randomized to medication or placebo. The time given in these plots is referred to the total period of the study. It can be observed that all the plots begin with survival probability 1 which means that no one has experienced the event in the beginning of the study. As the time passes, the survival curve tends to decrease because of the occurrence of events.

**Fig 1:**
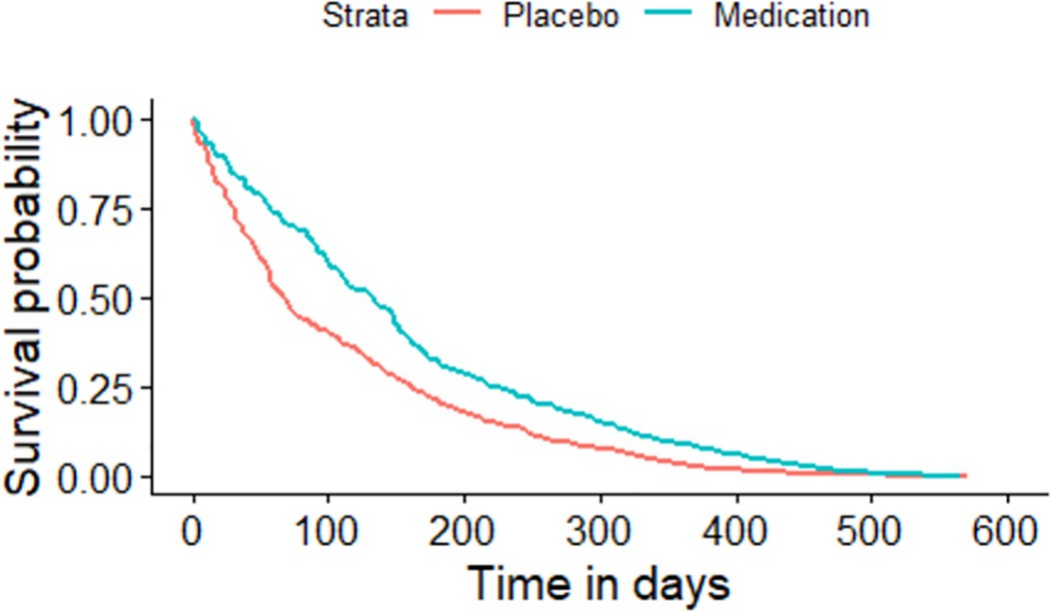
Kaplan-Meier Survival Curve for the entire dataset considering each observation as an individual.

**Fig 2:**
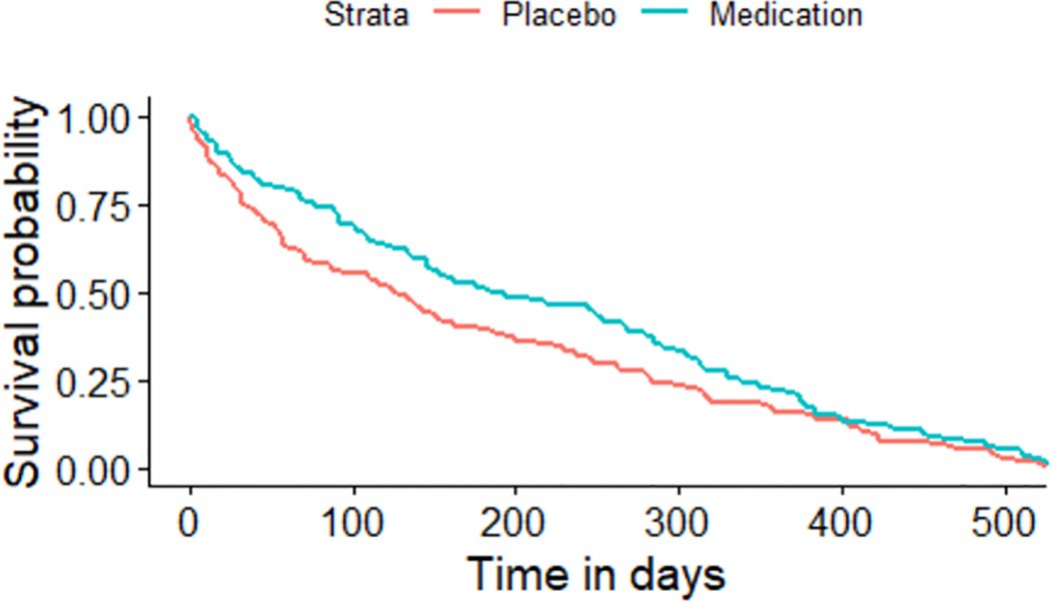
Kaplan-Meier Survival Curve for considering first event.

**Fig 3:**
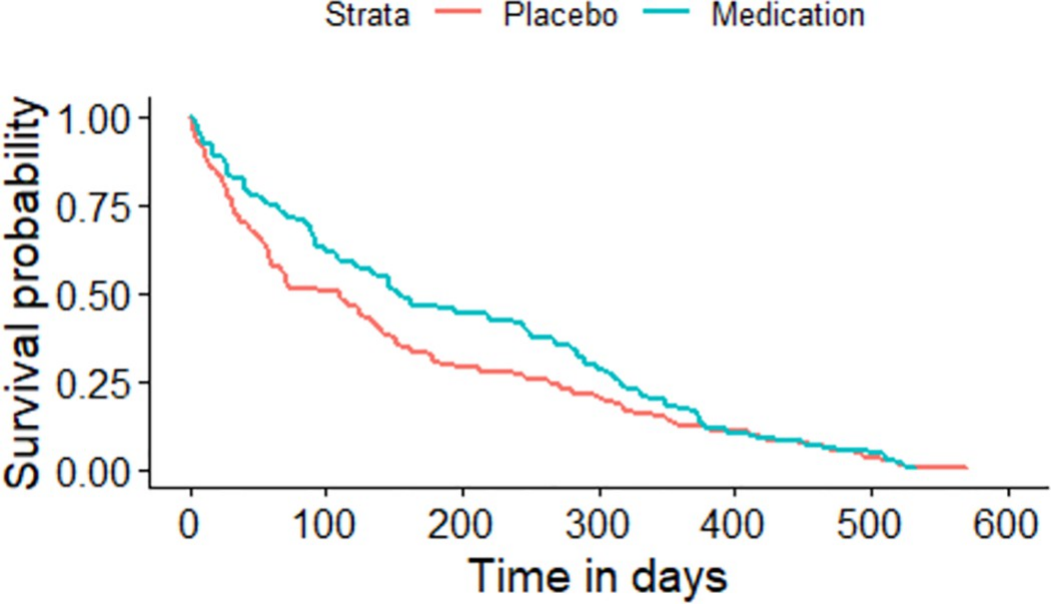
Kaplan-Meier Survival Curve for considering second event.

**Fig 4:**
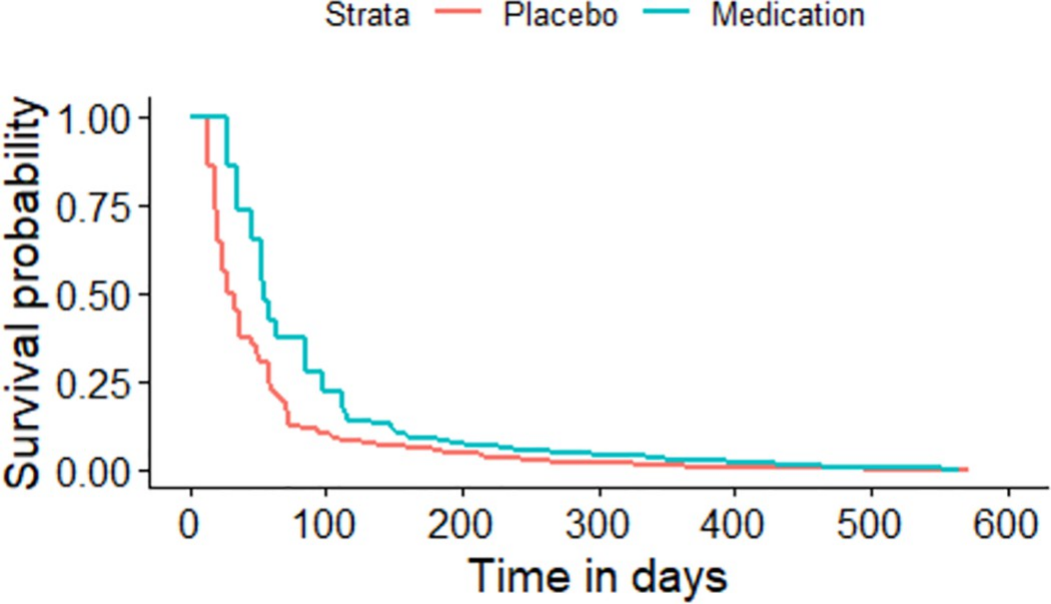
Kaplan-Meier Survival Curve for considering third event.

**Fig 5:**
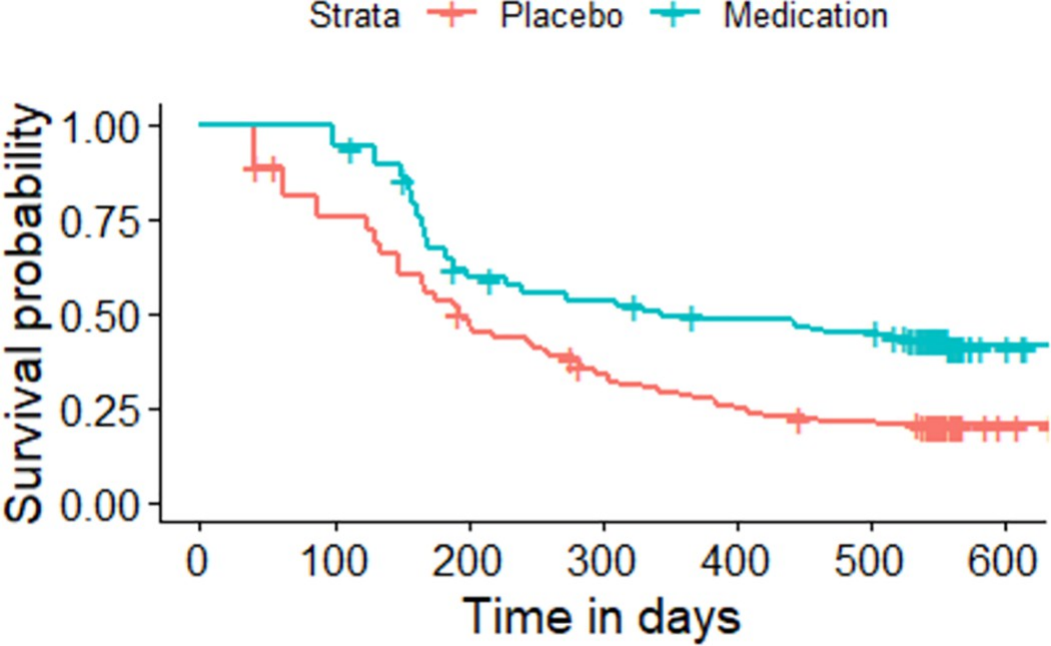
Kaplan-Meier Survival Curve for considering only fourth event.

**Fig 6:**
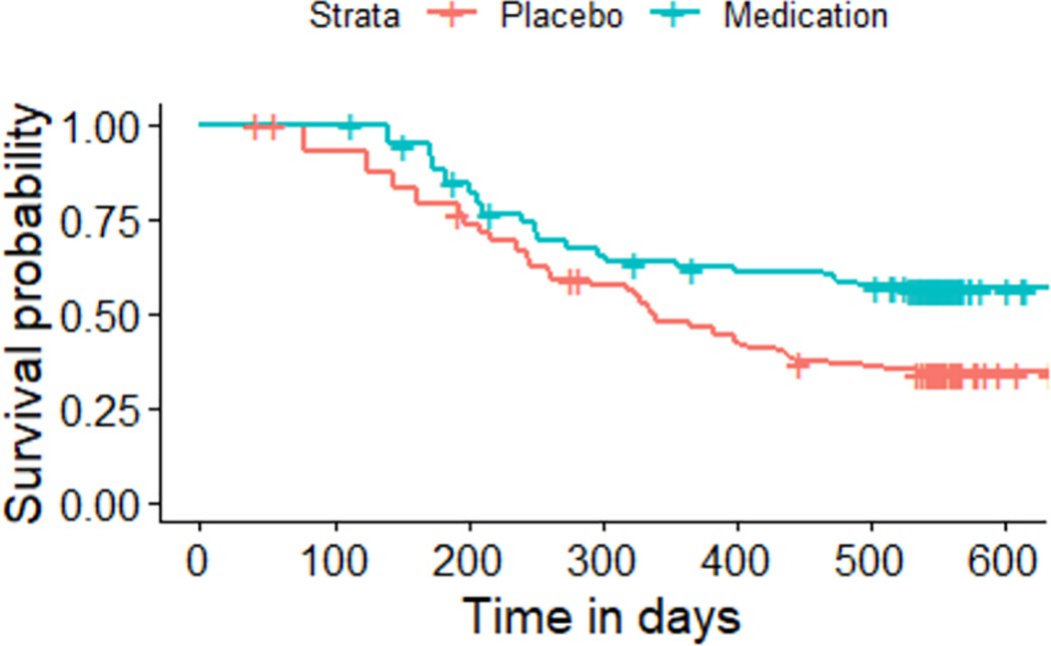
Kaplan-Meier Survival Curve for considering fifth event.

**Fig 7:**
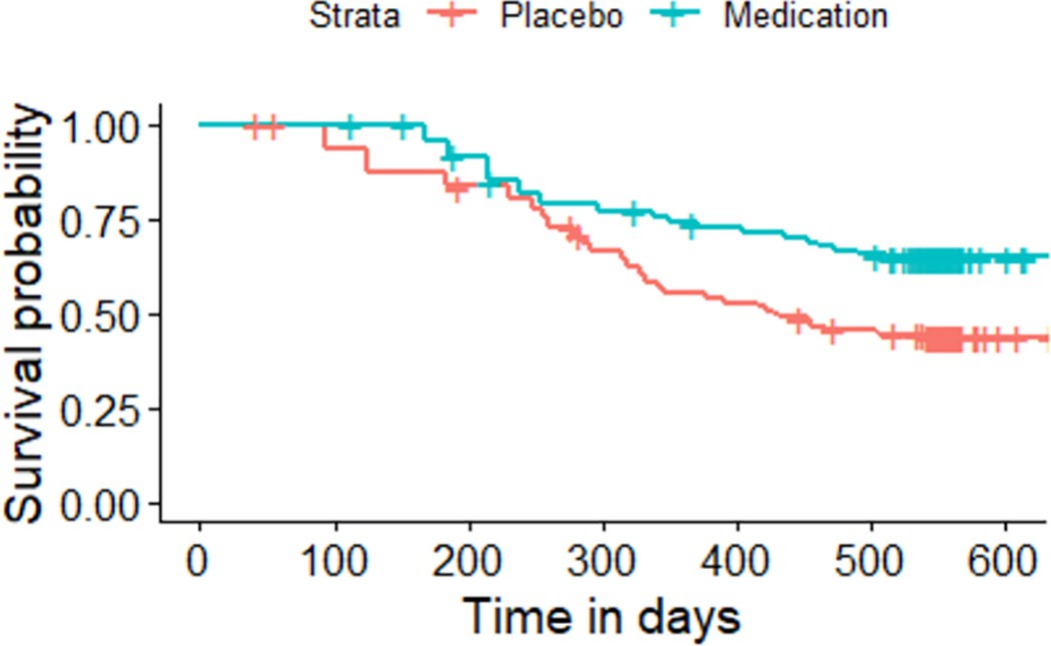
Kaplan-Meier Survival Curve for considering sixth event.

**Fig 8:**
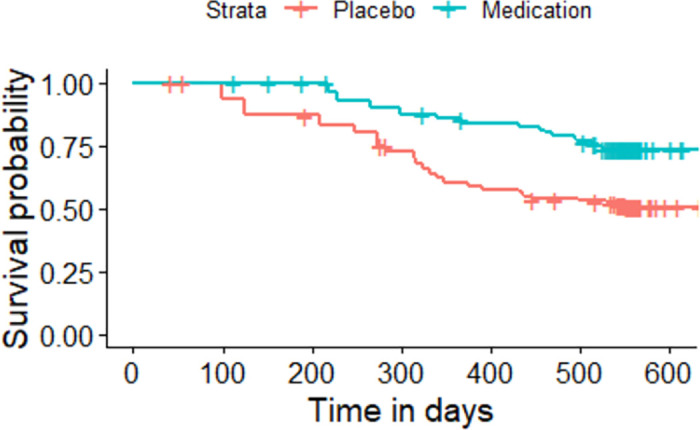
Kaplan-Meier Survival Curve for considering seventh event.

Fig 1 is based on the entire dataset ignoring that each observation in the dataset does not portray a single individual as most of the individuals have multiple entries due to recurrent events. The data is further split based on the order or sequence of the occurrence of events and Kaplan-Meier curves are plotted accordingly. For instance, Fig 2 shows only the first event experienced by all 232 children in the study. It’s Kaplan-Meier Survival Curves show the survival probability starting with 1 and ends with 0 since all the participants of the study have experienced the first asthma attack. In Figs 3–8, only the second event, third event, fourth event, fifth event, sixth event and seventh event are considered, respectively, taking all those individuals into account who are either censored or have experienced the second event. It can be observed from the given plots that when the number of asthma attacks for an individual increases on other hand the survival probability also increases. The reason is that different children experience different number of asthma attacks.

The detailed summary of the Cox model [17] considering calendar time based on the asthma patients data is given in Table 2.

**Table 2:** The estimates of various models based on calendar and gap time.

Model	coef	exp(coef)	se(coef)	z	*Pr*(> |*z*|)
**Cox-calendar**	-0.308	0.735	0.071	-4.337	1.44E-05
**Cox-first event**	-0.252	0.777	0.132	-1.903	0.057
**Cox frailty-calendar**	-0.302	0.739	0.123		
**Cox-gap**	-0.221	0.802	0.071	-3.1	0.002
**Cox frailty-gap**	-0.241	0.787	0.109		

The variable treatment (trt.w) is encoded a numeric vector; 0 for children randomized to placebo and 1 for those randomized to medication. The beta coefficient for treatment =—0.308 indicates that the children randomized to medication have lower risk of experiencing asthma attack compared to the children randomized to placebo. In other words the effect of treatment is large and the risk is relatively low. The exponential coefficient = 0.735 also called hazard ratios, give the effect size of covariates. For example, children randomized to medication reduce the hazard by a factor of 0.735 or 27 per cent.It can be seen that the variable treatment has highly statistically significant coefficient. In this case, the null hypothesis can not be accepted since, P value for all the three given tests (the likelihood ratio test, Wald test and score (logrank) test) are 0. It is worth mentioning that this is a wrong way to fit a model on such a dataset because it has been neglected that the data contains recurrent events or repeated outcomes. As mentioned earlier, total number of observations do not portray the number of children involved in the study. The data contain 1037 observations while there are 232 children involved in the study. It is because of the fact that number of asthma attacks varies among the children and most of them have experienced different number of asthma attacks. They have multiple entries in the dataset hence, the number of observations do not represent the number of children involved in the study.

The Cox model is fitted considering all the children involved in the study since all of them experience the first asthma attack. This model is not entirely wrong as it represents all the participants of the study but the prime concern is that a huge amount of data is lost as there are many children involved in the study who experience more than one asthma attack. From the output, the beta coefficient for treatment is equal to -0.252 which shows that the low risk is associated with the participants of the study. The hazard ratio is 0.777 which means the children randomized to medication reduces hazard by 22%.

The coefficient for Cox frailty-calendar model is -0.302 and the coefficient for Cox-calendar model is -0.308 which means the estimates for treatment effect and heterogeneity are nearly the same. On the other side, the coefficient for Cox frailty-gap model is -0.241 and the coefficient for Cox-gap model is -0.221 which implies that the treatment effect and heterogeneity are slightly smaller. In conclusion, the treatment effect is not largely affected by the elimination of the frailty term. Furthermore, adding a fertility term to the model increases standard error and less significant coefficients are obtained. It is concluded by stating that the use of gap and calendar times scales makes the interpretation of the data in different ways since, both the time scales model the data differently.

In the next section, we present the experiments conducted and the results obtained through the application of Landmark Analysis. Landmark Analysis, a well-established approach in survival analysis, allows us to generate dynamic predictions of survival and assess the prognosis of individuals at various time points during the follow-up period. By employing this methodology, we aim to investigate the predictive capabilities of the Landmark approach in our specific research context.

### 6.2 Landmark analysis

**Fig 9:**
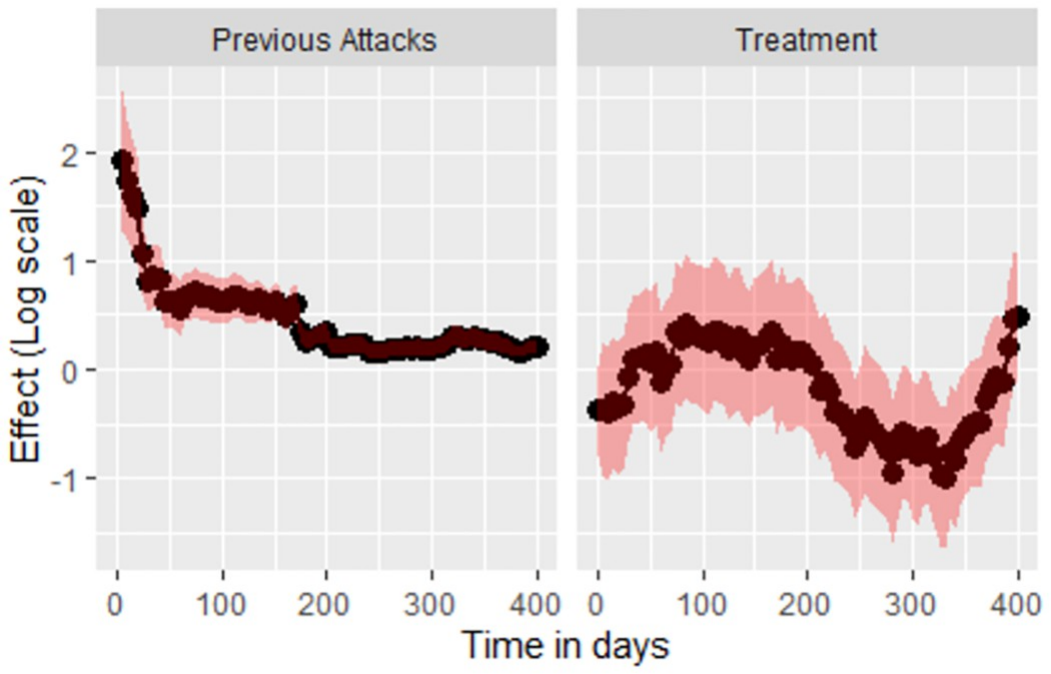
Treatment effect and Risk of experiencing asthma attack for each Landmark time point.

The first plot of the Fig 9 related to the treatment shows the dynamic treatment effects of the asthma prevention trial in young children. It can be seen that treatment effects varies with time. It can also be observed that the asthma patients are at high risk and the treatment effect is low from day 50 to day 200. Meanwhile the risk reduces and the treatment effect tends to be high from day 200 onwards until day 350. Eventually, the treatment effects drops again and remained low until the last Landmark time point 400. The second plot of Fig 9 related to the asthma attack shows the effect of previous attack. This plot basically shows that when the time goes by, the effect of previous attack reduces.

Table 3 shows the estimates for different covariates using super Landmark model 1 and super Landmark model 2. Super Landmark model 1 is stratified model where Landmark time points are chosen an equidistant grid from *s*_1_ = 0 to *s*_*L*_ = 400 days with distance 5. The resultant beta value for super Landmark model 1 is -0.247 which can be interpreted as the large treatment effect or the participants of the study are having low risk based on the given treatment. This outcome is obtained by using super Landmark model 1 utilizing the super prediction data set. It can also be observed that covariate (trt.w) value is statistically significant i.e., p value = 0.02 as shown in Table 3. The super Landmark model 2 is different than the super Landmark model 1 in such a way that the estimates of super Landmark 2 are obtained by adding a Landmark term to the model and by using unstratified analysis. The time functions chosen were *f*_1_(*s*)≡1, $f_{2}\left(s\right)=\frac{s}{150}$ and $f_{3}\left(s\right)={\left(\frac{s}{150}\right)}^{2}$. The beta value for super Landmark model 2 is -0.153 which means the risk is low or the treatment effect is large considering the super prediction dataset but the *trt*.*w* covariate is not statistically significant since the p value is greater than 0.05. Considering the beta values in super Landmark model 1 and super Landmark model 2, their difference is low and similarly their standard errors are almost similar but the *trt*.*w* covariate is not statistically significant in the case of super Landmark model 2. All other three variables *cnt*, *LM*1 and *LM*2 are statistically significant.

**Table 3:** The estimates for different covariates using super Landmark models.

Model	Factors	coef	exp(coef)	se(coef)	robust se	z	*Pr*(> |*z*|)
Super Landmark model 1	trt.w	-0.247	0.781	0.021	0.106	-2.33	0.02
Super Landmark model 2	trt.w	-1.53E-01	8.58E-01	2.18E-02	9.45E-02	-1.62	0.1
	cnts	1.30E-01	1.14E+00	2.77E-03	1.85E-02	7.05	1.80E-12
	LM1	-2.52E-12	1.00E+00	1.01E-01	2.80E-13	-9.02	< 2*e* − 16
	LM2	3.98E-13	1.00E+00	4.24E-02	8.27E-14	4.81	1.50E-06

In comparing our research to the study by [23], we identified certain commonalities and distinctions in our methodologies and findings. Both studies explore dynamic prediction of survival outcomes in the context of pediatric asthma patients, highlighting the significance of considering the evolving prognosis of young children over time.

However, there are notable differences in the approaches employed. Our research focuses on utilizing the Landmark method to obtain dynamic predictions, while the study by [23] adopts frailty models. These distinct modeling techniques can lead to varying results and interpretations. Furthermore, our study introduces specific modifications to the dataset, such as the inclusion of gap time variables and sequencing of events, to enhance the accuracy of our predictions. This methodological difference may influence the comparisons of the survival dynamics between the two studies. In short, by comparing our research to [23], we aim to provide a comprehensive understanding of the dynamic prediction of survival in pediatric asthma patients. By acknowledging the similarities and differences between our methodologies and findings, we contribute to the growing body of knowledge in this field.

## 7 Discussion

The different Landmark models utilize the data in different patterns. In the case of fitting simple Landmark model, the data is split into different Landmark points. In order to fit such a model, the data from a specific Landmark time point to a certain horizon is considered. In addition, truncation at Landmark point and administrative censoring at a horizon is required to get a sliding Landmark dataset. It ultimately provides dynamic predictions based on each Landmark point. The super Landmark models consider the super prediction dataset. The super Landmark model 1 is a stratified model which considers a grid of Landmark points and stacking them with stratification on the Landmark point. On the other side, the super Landmark model 2 is used by applying unstratified analysis with addition of Landmark term to the model. In Landmark super model 1 the predictions are obtained from stratum specific models while in Landmark super model 2 predictions are obtained for all *s* ∈ [*s*_1_, *s*_*L*_].

Fitting simple Landmark model to various sliding Landmark datasets provided different estimates during different time points of the study. It is observed that the treatment effect varies over time. With the help of such dynamic predictions the decision can easily be made regarding the continuation or stopping of particular treatment given to patients considering their prognosis. The treatment effects are estimated using different models such as Cox frailty-calendar model, Cox frailty-gap model, the Landmark super model 1 and Landmark super model 2. The estimates of all the models are having a negative beta value which means the treatment has a large effect on the asthma patients and their overall risk is relatively low. It is observed that the estimates obtained from Cox frailty-gap model and the super Landmark model 1 is almost the same i.e., -0.241 and -2.47 respectively. In super Landmark model 1 and super Landmark model 2, the difference between their respective beta value is low and similarly, their standard errors are almost similar but the *trt*.*w* covariate is not statistically significant in the case of super Landmark model 2 where its p value is 0.1. As concerned about the risk of experiencing the asthma attack, it remained high at the beginning of the study since, all the asthma patients were at high risk. With the passage of time the risk of experiencing asthma attack decreases and the reason could be that most of the participants in get censored after experiencing few asthma attacks while only few of them experienced high number of asthma attacks. The effect of previous asthma attack for different Landmark points are shown with the help of Landmark approach. It is observed that the effect of previous attack reduces as the time passes.

Howevere, Landmarking can be used for producing dynamically updated predictions of survival probabilities with time-dependent covariates in practice. Apart from this, Landmarking relies on strong assumptions about the time-varying covariate for validity that is why it may be impractical in the case of longitudinal biomarker measurements. On the side, Joint modeling could also be used because this method is quite flexibile in the case of time-dependent covariate, but may involve more modeling assumptions and is computationally expensive in general. Furthermore, this work can be extended by studying other novel outcomes as one of the main findings in future.

## 8 Conclusion

Determining dynamic effects of the covariates can be analyse through Landmark approach. This approach provides basis for the dynamic prediction of survival, considering time-to-event data. In conclusion, the treatment effect does not remain the same for long while it keeps varying from one Landmark time point to another. In addition, the effect of the previous attack reduces with the passage of time.
